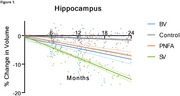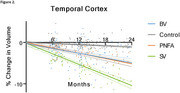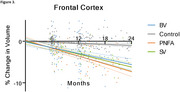# Advanced MRI Biomarkers and Deep Learning for Efficient Clinical Trials in Frontotemporal Dementia Subtypes

**DOI:** 10.1002/alz70862_109828

**Published:** 2025-12-23

**Authors:** Simone P Zehntner, Jean‐Philippe Coutu, Felix Carbonell, Alex P Zijdenbos, Barry J Bedell

**Affiliations:** ^1^ Biospective Inc., Montreal, QC Canada; ^2^ Biospective Inc, Montreal, QC Canada

## Abstract

**Background:**

Frontotemporal Dementia (FTD) encompasses a spectrum of neurodegenerative disorders characterized by progressive atrophy in the frontal and temporal lobes. These disorders manifest in three primary subtypes: behavioral variant FTD (bvFTD), semantic variant primary progressive aphasia (svPPA), and non‐fluent variant primary progressive aphasia (nfvPPA). Each subtype presents unique clinical and anatomical changes. Understanding these patterns is critical for early diagnosis, tracking disease progression, and designing effective clinical trials for potential disease‐modifying therapies. This study leverages advanced imaging techniques and automated processing pipelines to identify sensitive biomarkers and optimize clinical trial design.

**Methods:**

MRI data from the Frontotemporal Lobar Degeneration Neuroimaging Initiative (FTLDNI) were analyzed using the PIANO™ automated pipeline for volumetric and diffusion MRI (dMRI) analyses. Gray matter density, mean diffusivity (MD), and free water (FW) were assessed in 238 participants: 52 bvFTD, 32 nfvPPA, 35 svPPA, and 117 healthy controls. Sample size calculations were performed to estimate the number of participants required to detect a 60% reduction in brain atrophy and diffusion metrics over 6 to 24 months. Deep learning‐based segmentation, particularly for the hippocampus, enhanced reliability and reduced variability.

**Results:**

Distinct patterns of brain atrophy emerged across FTD variants as illustrated in Figure 1, with svPPA (green) exhibiting the most rapid progression, particularly in the hippocampus, temporal cortex, and amygdala, with up to 15% volume loss over 24 months. bvFTD (blue) primarily showed frontal and cingulate cortical changes, while nfvPPA (orange) demonstrated moderate, less localized changes. Sample size requirements were lowest for svPPA, with fewer than 35 participants per arm needed to detect therapeutic effects in key brain regions within 6 to 12 months. PIANO™‐based analyses demonstrated greater sensitivity and smaller sample size needs compared to other methods.

**Conclusion:**

This study highlights the utility of advanced imaging biomarkers in differentiating FTD subtypes and monitoring progression. The integration of volumetric and dMRI metrics, along with deep learning segmentation, offers precise, early detection of changes, thereby reducing sample size requirements and enabling cost‐effective, efficient clinical trials. Furthermore, this approach offers insights into the unique spatial and temporal progression patterns of each FTD subtype, paving the way for personalized intervention strategies.